# Self-assessment, attitude and perception of interprofessional learning in hospital acquired infection control practices among health professionals in Klang Valley, Malaysia

**DOI:** 10.1186/s12909-021-02610-1

**Published:** 2021-04-26

**Authors:** Saraswathy Thangarajoo, A. M. Rosliza, Sivalingam Nalliah, Jalina Karim, Shamarina Shohaimi, S. Ramasamy, S. Amin-Nordin

**Affiliations:** 1 Department of Medical Microbiology, Faculty of Medicine and Health Sciences, Universiti Putra Malaysia, Serdang, Malaysia; 2 International Medical College, Subang Jaya, Malaysia; 3 Department of Community Health, Faculty of Medicine & Health Sciences, Universiti Putra, Serdang, Malaysia; 4 Department of Obstetrics and Gynaecology, Clinical Sciences, International Medical University, Seremban, Malaysia; 5 Department of Nursing, Faculty of Medicine, Universiti Kebangsaan Malaysia, Bangi, Malaysia; 6 Department of Biology, Faculty of Science, Universiti Putra Malaysia, Serdang, Malaysia; 7 Department of Psychology, International Medical University, Bukit Jalil, Kuala Lumpur, Malaysia

**Keywords:** Interprofessional, Learning, Self-assessment, Perception, Attitude, Doctor, Nurse

## Abstract

**Background:**

Interprofessional learning (IPL) is a key challenge in Malaysia in incorporating the current profession-specific medical education into the interprofessional learning curriculum. Safe practices would be enhanced with improved collaboration among the health professionals when they learn with, from, and about each other. The main objective of this study was to determine the significant differences in self-assessment, attitude, and perception of interprofessional learning among doctors and nurses in a teaching hospital in Klang Valley, Malaysia. The second objective was to determine if there is any significant difference in the selected demography factors, mean and total scores between doctors and nurses in self-assessment, attitude, and perceptions of IPL aspects.

**Methods:**

A total of doctors (39) and nurses (37) were recruited for an interventional study on the interprofessional learning approach on hospital acquired infection control. The participants responded to the University of West England Interprofessional (UWEIP) questionnaire at baseline consisting of four dimensions in IPL aspects; Self-assessment on communication and teamwork skills (CTW), interprofessional learning (IPL), interprofessional interaction (IPI), and interprofessional relationship (IPR). The Cronbach alpha value for the total questionnaire was established at 0.79.

**Results:**

The majority of doctors scored positive in CTW, IPL, IPR, and neutral in IPI. Nurses’ also recorded the highest positive scores in CTW, IPL, and IPR, and neutral in IPI. Negative scores were found in CTW and IPI. A significant difference was revealed between doctors and nurses in IPL attitude; *p* = 0.024 and there was no significant difference in other dimensions (*p* > .05). Results also found a significant difference between participants’ and non-participants of IPL training sessions; *p* = 0.009.

**Conclusions:**

This study revealed the infusion of interprofessional learning training among the health professionals displayed better self-assessments, attitudes, and perceptions towards collaborative practices.

## Background

Parallel to the global medical education wave of “Learning together to work together for Health’ [1], interprofessional learning (IPL) is a key issue to be addressed in Malaysia.

Interprofessional learning (IPL) refers to “occasions when two or more professionals learn with, from and about each other to improve collaboration and the quality of care”. It promotes interdisciplinary collaboration and teamwork, reducing barriers and misconceptions among healthcare providers, resulting in better management of the patient [2].

Interprofessional Education Collaborative Experts Panel in 2011, outlined four IPL core competency domains namely, ethics and values, roles and responsibilities, interprofessional (IP) communication and teams, and teamwork. Six disciplines which consist of dentistry, nursing, medicine, osteopathic medicine, pharmacy, and public health were included in this initiative [3]. The report also guided in preparing future health professionals for collaborative safe patient-centered care. Since then, the core competencies were broadly disseminated to guide curriculum development in medical education.

In 2016, IPEC organized the four competencies within a singular domain of IP collaboration to better improve the ‘Triple Aim’ which are the patient experience of care, health of population, and reduction of cost of health care. Furthermore, it was also outlined hospital- acquired infection control practices as one of the aspects to begin IPL among health professionals [4].

The establishment of professional collaboration between doctors and nurses gained attention ever since the publication of the hallmark article by Dr. Stein Leonard in 1967 [5].

These professions demand intense mutual respect and cooperation between the team members. The stereotype attitude towards nurses as the handmaiden of male doctors creates serious resistance to ineffective communications between them as the doctors seek information about patient progress from nurses. The doctor-nurse game illustrated nurses need to be bold and have the initiative to make momentous recommendations, but they need to appear passive [5].

Over the years, the progression in nursing became significant as it evolved as a profession with its own autonomy and not merely the handmaiden of doctors and taken professional roles in patient care. In 1990, Stein, Watts, and Howell revisited the doctor-nurses ‘game’ and they recognised the evolution of nursing practice as a profession parallel to other health professionals [6]. However, since then, the author’s opinion became debatable with many studies that have reported the game is still been played. There is a need for greater dialogue as all health professionals are experts in their field and need to collaborate for the good of the patients.

Doctors and nurses as students build their own professional identities, as their training program is different with emphasis varying. New relationships are developed when they enter the profession and share common core values, knowledge, and skills. The “professional silos” often foster interactions based on power, competition, and hierarchies, resulting in insufficient preparation for collaboration among them. They are unrealistically expected to function in collaborative health care teams. Interprofessional learning can introduce shared learning early and help prepare learners for collaborative practice [7]. Furthermore, advances in IPL competencies fostered moving beyond profession-specific educational efforts to engage students of different professions in interactive learning [8].

The main objective of this study was to evaluate the current perception of readiness in interprofessional learning aspects among doctors and nurses in hospital acquired infection control practices. The second objective was to determine if there is any significant difference in the selected demography factors, mean and total scores between doctors and nurses in self-assessment, attitude, and perceptions of IPL aspects.

### Barriers in interprofessional learning

Training healthcare professionals for interprofessional work has an important role in increasing the quality of services rendered to the patient and community at large. Hierarchical questions often result in significant barriers to patient safety and disconnect among professions. Healthcare professions also need to change the way they relate to one another before they can work in collaborative teams [9]. The most effective period to introduce the principles of IPL in the curriculum for training healthcare professionals is still debatable and varies across different institutions [10].

Moreover, organisational hierarchy such as seniority often creates obstacles to the formation of interprofessional collaboration among doctors and nurses. Nurses find that junior doctors are generally more open to suggestions, showing non-competitive roles in patient care [9]. Often junior doctors depend on nurses for essential sources of information as they are prescribing treatment while the senior doctors finalise medical decisions. It is not uncommon to see differences in opinion and decision-making challenges between senior physicians and experienced nurses which is less apparent with junior doctors [11].

This relationship is less apparent between junior doctors and nurses caring for the patient [12]. In a qualitative study by Long et al. on medical dominance in interprofessional collaboration, doctors’ opinions were often dominant, and non-doctors’ opinions were often muted. Doctors’ time was appreciated over nurses’ time, both within the clinical team and in the broader hospital environment. The doctors’ power of speech had more authority and autonomy than nurses [13].

### Significance of the interprofessional learning study

Doctors and nurses in the current study had undergone their training in traditional profession–specific programs. Little evidence is garnered to demonstrate that they possess formal exposure to IPL in their training. Clearly, there is a need to be formally trained in IPL when supervising medical and nursing students during their formative years in order to minimize gaps in clinical practice in IPL. Previous poor experiences within the healthcare team can reinforce negative beliefs about the value of other health professionals to learners [14].

In spite of the growth in the subject of IPL as reported in the literature [15], a search of Malaysian literature showed that there are few studies concerning to aspects of IPL as a medical educational strategy. The case for instructions in IPL is borne by Gambino et al.’s study which shows evaluations for courses on interprofessional team-based learning between medical and nursing students, have been very positive [16]. In fact, early exposure to IPL will allow students to develop their own professional identity together with the knowledge, skills, attitudes, and behaviour that will expedite future collaboration [17].

The hospital where this study was conducted is providing clinical placement for students from various fields. The doctors and nurses are observed by these students as role models. This descriptive comparative study aimed to identify the aspects of IPL among doctors and nurses in the pre-interventional stage of a doctorate study to report on baseline findings.

## Methods

### Research design

This was a cross-sectional study carried out from June 2018 to January 2019.

### Research setting

This study was conducted in Hospital Serdang, which is an affiliated teaching hospital for Universiti Putra Malaysia in Klang Valley, Malaysia. Serdang Hospital is a 620 bedded government-funded multi-specialty hospital located in the district of Sepang in the state of Selangor, Malaysia. It is designated as the tertiary referral center for nephrology, urology, and cardiothoracic surgery. Permission to conduct this study was granted by the Clinical Research Centre of Hospital Serdang. The heads of the departments were approached to recruit participants on a voluntary basis. Purposive sampling was used to recruit participants from medical, surgical, pediatric, emergency, and intensive care units. All participants signed the consent form on a voluntary basis.

### Participants

#### Sample size estimation

Sample size estimation was done using Lehr’s equation: [18].

The formula for the sample size to compare two population means, μ0 and μ1, with common variance ∂^2^ is:

$$ \mathrm{N}=\frac{\left[\left(\mathrm{r}+1/\mathrm{r}\right)\right]{\left[\mathrm{Z}\;1\hbox{-} \upalpha /2+\mathrm{Z}\;1\hbox{-} \upbeta \right]}^2}{{\left[\left(\upmu 0\kern0.5em \hbox{-} \kern0.5em \upmu 1\right)/\partial \right]}^2} $$

Therefore, $$ {\displaystyle \begin{array}{l}\mathrm{N}=\frac{2{\left[1.96+0842\right]}^2}{\Omega^2/{\partial}^2}=\frac{15.68}{2/{\partial}^2}\\ {}=16/{\Delta}^2\end{array}} $$

α Significance level set at 5%. Hence, Z1-α/2 is 1.96.

1 - β power set at 80%. Hence, Z 1-β is 0.842

Ω Test value of the difference between two means.

∂ Anticipated SD of the response variable/parameter.

Δ Standardized Difference is the treatment difference to be detected in units of the standard deviation = [(μ0 - μ1) / ∂] = Ω2 / ∂2.

r Ratio of interventions against the controls = 1.

Here, the design effect is the anticipated standard deviation of the response variable on the intervention.

Based on Flude et al. (2016), mean knowledge in infection control has increased from pretest; 84.95% (Mean = 5.75; SD 1.09; *n* = 189) to post-test; (Mean = 6.25; SD =0.81; *n* = 106). In this study, the mean difference has been set at 1.00 and SD = 1.5 [19]. Eighteen (18) doctors (18); and eighteen (18) nurses are needed for each group (based on Lehr’s formula calculation) and the means score is based on a previous study by Flude et al. (2016) [19]; (Mean = 6.25, SD = 109). However, providing an additional 50% for attrition rate [20] in order to estimate the loss to follow up at 3 months post-intervention assessment, the total sample size of 54 for each.

intervention and control groups will be fixed with equal numbers of doctors (27) and nurses (27) in each group, totaling 108 participants. The tool used to calculate this sample size is as retrieved from:

http://www.openepi.com/SampleSize/SSMean.htm

### Inclusion criteria

The inclusion criteria include consist of doctors and nurses working in the high risk and medium risk areas in hospital acquired infections. Due to the constraints in getting the number of participants, medical and surgical wards were also included.

### Exclusion criteria

Doctors and nurses who are not consenting to the study, maternity leave and annual leave on days of data collection were excluded in this study.

### Instruments

The University of West England (UWE) Interprofessional (IP) questionnaire was selected as the instrument to be used. A pool of professionals including nursing faculty was involved in the validation of the questionnaire. The questionnaire consists of four dimensions measures the construct of interprofessional learning:

i. Communication and Teamwork (CTW)
ii. Interprofessional Learning (IPL)
iii. Interprofessional Interaction (IPI)
iv. Interprofessional Relationship (IPR)

The objective of the questionnaire is to measure self-assessment on communication and teamwork skills, attitude and perceptions towards interprofessional learning and interaction, and on relationships among disciplines. The CTW, IPL and IPR were developed by Pollard et al. (2004). The reliability coefficients measured using Cronbach alpha obtained were 0.76 (*n* = 813), 0.84 (*n* = 836) and 0.82 (*n* = 825) respectively [21]. The IPR dimension was tested by Pollard et al.(2005) and the overall Cronbach alpha was 0.71 (*n* = 694) [22]. The stability of the questionnaire was tested using a test-retest method. Permission has been granted by Dr. Katherine Pollard to use the questionnaire for this study.

Concurrent validity was established for each dimension. The teamwork and communication scale responses were compared to those of the Interpersonal Communication Competence Scale [23], and responses to the scale concerning interprofessional learning with those to the Readiness for Interprofessional Learning Scale respectively [24]. The Interprofessional Interaction Scale was supported by qualitative data from students’ interview on perception towards interprofessional interaction in healthcare [21] and Interprofessional Relationships Scale was compared with the Interdisciplinary Education Perception Scale by Leucht et al. (1990) [25] as reported by Pollard et al. in 2005 [22].

Three (3) expert panels consisting of two medical specialists with interprofessional teaching and clinical expertise and one Deputy Director of Nursing from the Nursing Board Malaysia with vast clinical expertise, validated the contents using a 4-point relevance scale [26]. The three experts scored ‘Very relevant’ to all the 35 items and no changes were made to the items. The questionnaire was piloted in the local settings among 15 doctors and 15 nurses. Negative items were recoded as recommended by the developer e.g. the third item in the CTW dimension ‘I have difficulty in adapting my communication style (oral and written) to situations and audiences’. The scores are then summed to produce a composite score. The Cronbach alpha for the 4 subscales was CTW (.552), IPL (.816), IPI (.675) and IPR (.775). The overall Cronbach reliability with a total of 35 items was established at .793.

The scores assigned for each dimension to categorise the achievements indicating positive, neutral and negative are as follows:

**i. Self- assessment on Communication and teamwork (CTW)**

- Nine statements on self-assessment of communication and teamwork skills were tested in the Communication and Teamwork (CTW) dimension. A 4-point Likert scale was scored from 1 (strongly agree) to 4 (strongly disagree). The neutral point was omitted as all participants are presumed to have experience in communication and group work at an informal level. The maximum score for this scale is 36, while the minimum is 9. Scores from 9 to 20, 21–25, and 26–36 are considered to indicate positive, neutral and negative attitudes respectively towards self-assessment of communication and teamwork.

**ii. Attitude in interprofessional learning (IPL) and perception in interprofessional interaction (IPI)**

- Five-point Likert scale was used; 1 (strongly agree) to 5 (strongly disagree). For the Interprofessional Learning (IPL) and Interprofessional Interaction Scales (IPI), scores from 9 to 22, 23–31, and 32–45 indicate positive, neutral, and negative attitudes respectively towards interprofessional learning and perceptions of interprofessional interaction (both these scales have a maximum score of 45 and a minimum of 9).
- The IPL dimension consists of 9 statements on attitude towards interprofessional learning. The statements tested how doctors feel about working together with nurses and vice versa.
- The IPI dimension consists of 9 statements on the perception of interprofessional interaction between doctors and nurses [20].

**iii**. **Attitude in interprofessional relationship (IPR)**

- The Interprofessional Relationships dimension has a maximum score of 40 and a minimum of 8. Scores from 8 to 20, 21–27, and 28–40 indicate positive, neutral, and negative attitudes respectively towards the respondent’s own interprofessional relationships.

The IPR scale tested on attitudes towards the relationship between doctors and nurses with 3 statements from their own discipline and 5 statements from other disciplines [22]. The IPL data was collected by face-face administration among participants in a study briefing session.

### Data analysis

Data analysis was carried out in two phases. First, a descriptive analysis was done, using frequency and percentage for sociodemographic data. Total scores were tabulated on the four dimensions results. Subsequently, the scores were compared by means between doctors and nurses to determine the significant difference in the scores. Homogeneity of variances and the normality of the distribution were tested by calculating skewness and kurtosis. The data were normally distributed and variances in the groups were homogeneous. Parametric tests were used in the statistical analysis.

An independent t-test was used to determine the significant difference between gender and participants and non-participants of IPL sessions. One-way ANOVA was carried out to assess the significant difference in age, highest qualifications, and years of practice. Statistical analysis was conducted using SPSS version 23.0 (SPSS Inc.) and a *p* < 0.05 was adopted as a decision criterion. Internal consistency measured using Cronbach’s alpha was 0.79(*n* = 76), Therefore the questionnaire satisfies conditions for adequate reliability of 0.7 [27].

## Results

The total number of participants in this study consist of 39 doctors and 37 nurses (Table 1). Most of the doctors (94.9%) and nurses (45.9%) are from the age group of 25–30 years. Doctors consist of 43.6% males and 56.4% females, whereas the majority of nurses were females. Nurses (40.5%) attended IPL training as compared to doctors (15.4%).

**Table 1:** Description of analysis on demographic data

	Doctors (*n* = 39) n (%)	Nurses (*n* = 37) n (%)
**Age (years)**		
25–30	37 (94.9)	17 (45.9)
31–35	2 (5.1)	13 (35.1)
36–40		4 (10.8)
40 and above		3 (8.1)
**Gende**r		
Male	17 (43.6)	2 (5.4)
Female	22 (56.4)	35 (94.6)
**Highest qualification**		
MBBS	39 (100)	
Diploma in Nursing		34 (91.9)
Bachelor of Nursing		3 (8.1)
**Discipline working**		
Surgical	20 (51.3)	9 (24.3)
Medical	6 (15.4)	9 (24.3)
Paediatric	5 (12.8)	2 (5.4)
Intensive Care Unit	1 (2.6)	9 (24.3)
Emergency (ED)	7 (17.9)	8 (21.6)
**Years of Practice**		
1–5	39 (100)	14 (37.5)
6–10		15 (40.5)
11–15		5 (13.5)
16 and above		3 (8.1)
**IPL Training** **Attendees**		
Yes	6 (15.4)	15 (40.5)
No	33 (84.6)	22 (59.5)

Table 2 describes the analysis of individual items across two dimensions, self-assessment on communication and teamwork (CTW) and interprofessional learning (IPL). Nine items were administered in each dimension. In CTW, the highest agreement was seen in item number 5 on their comfort in working with other staff in a group, with doctors responded, ‘strongly agree” (38.5%) and ‘agree’ (48.7%) and; nurses recorded 56.8% ‘strongly agree’ and 35.1% ‘agree’. The highest disagreement was revealed on item number 7; ‘I feel uncomfortable putting forward my personal opinions in a group’. Doctors scored 43.6% (‘disagree’) and 5.1% (‘strongly disagree’); compared to nurses 35.1% (‘disagree’) and 5.4% (‘strongly disagree’).

**Table 2:** Analysis of items on Communication and Teamwork and Interprofessional Learning

Item Communication and Teamwork	Strongly Agree *(*%)		Agree (%)		Neutral (%)		Disagree (%)		Strongly Disagree (%)	
	D	N	D	N	D	N	D	N	D	N
1.I feel comfortable justifying recommendations /advice face to face with more senior people	33.3	27	35.9	48.6	–	–	28.2	24.3	2.6	–
2.I feel comfortable explaining an issue to people who are unfamiliar with the topic	25.6	16.2	48.7	48.6	–	–	25.6	32.4	–	2.7
3.I have difficulty in adapting my communication style (oral and written) to particular situations and audiences	2.6	13.5	59	54.1	–	–	33.3	32.4	5.1	–
4.I prefer to stay quiet when other people in a group express opinion that I don’t agree with	10.3	18.9	51.3	64.9	–	–	25.6	16.2	12.8	–
5.I feel comfortable working in a group	38.5	56.8	48.7	35.1	–	–	7.7	8.1	5.1	–
6.I feel uncomfortable putting forward my personal opinions in a group	12.8	32.4	38.5	27	–	–	43.6	35.1	5.1	5.4
7.I feel uncomfortable taking the lead in a group	15.4	24.3	41	37.8	–	–	35.9	32.4	7.7	5.4
8.I am able to become quickly involved in new teams and groups	20.5	21.6	48.7	43.2	–	–	30.8	35.1	–	–
9.I am comfortable expressing my own opinions in a group, even when I know that other people don’t agree with them	17.9	13.5	41	45.6	–	–	41	32.4	–	8.1
**Interprofessional Learning**										
10.My skills in communicating with patients/clients would be improved through learning with doctors/ nurses	35.9	24.3	51.3	51.4	7.7	21.6	5.1	–	–	2.7
11.My skills in communicating with doctors/nurses would be improved through learning with them.	48.7	32.4	43.6	45.9	5.1	18.9	–	2.7	2.6	–
12.I would prefer to learn only with peers from my own profession	30.8	32.4	33.3	45.9	10.3	10.8	15.4	5.4	10.3	5.4
13.Learning with doctors/nurses is likely to facilitate subsequent working professional relationships	41	21.6	48.7	43.2	10.3	24.3	–	2.7	–	8.1
14.Learning with doctors/nurses would be more beneficial to improving my teamwork skills than learning only with my peers.	33.3	13.5	48.7	48.6	12.8	24.3	2.6	2.7	2.6	10.8
15.Collaborative learning would be a positive learning experience for all doctors and nurses	46.2	40.5	43.6	43.2	7.7	13.5	2.6	–	–	2.7
16.Learning with doctors/nurses is likely to help to overcome stereotypes that are held about the different professions	43.6	16.2	48.7	51.4	5.1	29.7	2.6	2.7	–	–
17.I would enjoy the opportunity to learn with doctors/nurses	33.3	29.7	56.4	56.8	10.3	13.5	–	–	–	–
18.Learning with doctors/nurses is likely to improve the service for patient/client	43.6	27	48.7	56.8	7.7	16.2	–	–	–	–

The IPL dimension measured on 9 items pertaining to attitudes on learning together between doctors and nurses. The highest agreement among doctors and nurses are seen in item number 18; ‘Learning with doctors/nurses is likely to improve the service for patient’. The doctors scored 43.6% (‘strongly agree’) and 48.7% ‘agree’. The nurses scored lower comparatively as 27% (‘strongly agree’) and 56.8% ‘agree’.

Table 3 shows the analysis of interprofessional interaction (IPI) and interprofessional relationship (IPR) dimensions. Nine items were measured in IPI. The highest agreement scores were among nurses on item number 23; ‘All members of medical and nursing professions have equal respect for each discipline’ The nurses revealed the highest agreement on this item with 35.1% ‘strongly agree’ and 43.2% ‘agree’. In the IPR dimension, eight items were tested. Item number 44 on; ‘I lack confidence when I work with people from doctors/nurses’ disciplines’ scored the highest agreement among doctors. They scored 46.2% (‘strongly agree’) and 48.7% ‘agree’ but nurses recorded only 24.3% (‘strongly agree’), 21.6% (‘agree’).

**Table 3:** Analysis of Interprofessional Interaction and Interprofessional relationship Dimensions

Item Interprofessional Interaction Scale	Strongly Agree *(*%)		Agree (%)		Neutral (%)		Disagree (%)		Strongly disagree (%)	
	D	N	D	N	D	N	D	N	D	N
19.Doctors and nurses have stereotyped views of each other.	5.1	–	5.1	13.5	33.3	18.9	38.5	43.2	17.9	24.3
20.The line of communication between all members of the doctors and nursing professions is open	20.5	35.1	35.9	37.8	28.2	13.5	10.3	13.5	5.1	–
21.There is a status hierarchy in health care that affects relationships between doctors and nursing professionals.	2.6	13.5	17.9	10.8	25.6	16.2	43.6	37.8	10.3	21.6
22.Doctors and nursing professionals are biased in their views of each other.	2.6	10.8	15.4	18.9	46.2	18.9	23.1	37.8	12.8	13.5
23.All members of medical and nursing professions have equal respect for each discipline.	25.6	35.1	30.8	43.2	20.5	8.1	17.9	10.8	5.1	2.7
24.It is easy to communicate openly with people from other health care disciplines.	7.7	21.6	43.6	43.2	33.3	18.9	12.8	16.2	2.6	–
25.Not all relationships between medical and nursing professionals are equal	–	2.7	2.6	2.7	43.6	21.6	41	43.2	12.8	29.7
26.Doctors and nursing professionals do not always communicate openly with one another.	7.7	13.5	17.9	16.2	30.8	21.6	38.5	27	5.1	21.6
27.Doctors and nursing professionals are not always cooperative with one another	12.8	24.3	17.9	24.3	30.8	21.6	33.3	18.9	5.1	10.8
**Interprofessional Relationship Scale**										
28.I have an equal relationship with peers from my own professional discipline	28.2	21.6	48.7	40.5	20.5	29.7	2.6	5.4	–	2.7
29.I am confident in my relationships with my peers from my own professional discipline	23.1	32.4	59	45.9	15.4	13.5	2.6	8.1	–	–
30.I have a good understanding of the roles of doctors/nurses’ professionals.	17.9	16.2	43.6	48.6	33.3	32.4	5.1	2.7	–	–
31.I am confident in my relationships with people from doctors/nurses’ disciplines	12.8	16.2	61.5	51.4	20.5	27	5.1	5.4	–	–
32.I am comfortable working with people from doctors/nurses disciplines.	25.6	21.6	59	37.8	12.8	35.1	2.6	5.4	–	–
33.I feel that I am respected by people from doctors/nurses’ disciplines	20.5	21.6	38.5	35.1	30.8	35.1	10.3	5.4	–	2.7
34.I lack confidence when I work with people from doctors/nurses’ disciplines.	46.2	24.3	48.7	21.6	5.1	32.4	–	21.6	–	–
35.I am comfortable working with people from own professional discipline	12.8	48.6	59	35.1	25.6	10.8	2.6	2.7	–	2.7

As displayed in Table 4, the majority of the participants scored positively on self-assessment of communication and teamwork (CTW) skills with higher scores among nurses 70.3% [Mdn = 19; 95% CI (16.5, 21.5)] compared to doctors 59% [Mdn =19, 95% CI (17, 22)]. Nurses scored 11.3% more positive than doctors. In interprofessional learning dimension, most of the doctors 92.3% [Mdn = 17, 95% CI (12, 19)] recorded positive attitude scores compared to nurses only 83.8% [Mdn = 18, 95% CI (16, 20)]. This finding shows doctors scored 8.5% more positive attitude towards interprofessional learning as compared to nurses.

**Table 4:** Analysis of scores for aspects on interprofessional learning

Dimension	Indication scores						Scores					
	Positive (9–20)		Neutral (21–25)		Negative (26–36)		Max (36)		Min (9)		Median (Range)	
	(%)		(%)		(%)							
Communication and Teamwork Scale (CTW)	D	N	D	N	D	N	D	N	D	N	D	N
	23 (59)	26 (70.3)	14 (35.9)	10 (27)	2 (5.1)	1 (2.7)	27	28	14	9	19 (13)	19 (19)
Interprofessional Learning Scale (IPL)	Positive (9–22) f(%)		Neutral (23–31) f (%)		Negative (32–45) f (%)		Max (45)		Min (9)		Median (Range)	
Scores	31 (92.3)	31 (83.8)	3 (7.7)	6 (6.2)	Nil	Nil	28	28	9	10	17 (19)	18 (18)
Interprofessional Interaction (IPI) Scale												
Scores	6 (15.4)	8 (21.6)	25 (64.1)	19 (51.4)	8 (20.5)	10 (27)	41	37	16	13	27 (25)	27 (24)
	Positive		Neutral		Negative		Max (40)		Min (8)			
Interprofessional Relationship Scale (IPR)	8–20		21–27		28–40							
Scores	33 (84.6)	26 (70.3)	6 (15.4)	11 (29.7)	Nil	Nil	23	26	11	8	18 (12)	19 (18)

Note; *D* Doctors, *N* Nurses, *Mdn* (Median); *f* Frequency

Interprofessional interaction dimension revealed doctors showed higher neutral perception, 64.1% [Mdn = 27, 95% CI (24, 31)] compared to nurses 51.4% [Mdn = 27, 95% CI (23, 32.5)]. This finding showed 6.2% of nurses have a more positive perception towards interprofessional interaction as compared to doctors. In interprofessional relationship (IPR) dimension, 84.6% doctors [Mdn = 18, 95% CI (15, 20)] revealed positive attitude compared to 70.3% among nurses [Mdn = 19, 95% CI (14, 21)]. This finding showed doctors revealed 14.3% higher positive attitude than nurses in IPR.

### Mean description of the dimensions

Table 5 shows the tabulation of t values for an independent t-test across four dimensions of IPL. The mean scores of the IPI are the highest among the 4 dimensions, whereas IPL has the lowest mean scores. Levene’s test is not violated, indicating the data are homogeneously distributed across both groups. There was no significant difference between doctors and nurses in CTW; t (74) = 1.200, *p* = 0.234, IPI; t (74) = .644, *p* = .522 and IPR; t (74) = −.298, *p* = .766. Whereas, a significant difference was shown in the IPL dimension between doctors and nurses; t (74) = − 2.305, *p* = 0.024, wherein nurses showed a higher attitude toward IPL.

**Table 5:** Comparison of mean in aspects on interprofessional learning dimensions among doctors and nurses

Dimension	Mean Score ± SD among Doctors	Mean Score ± SD among Nurses	F	t	df	*p*-value
Communication and teamwork	2.19 (.386)	2.08(.454)	.094	1.200	74	.234
Interprofessional Learning	1.80 (.534)	2.06(.440)	1.744	−2.305	74	.024
Interprofessional Interaction	3.06(.585)	2.97(.669)	.886	.644	74	.522
Interprofessional Relationship	2.16(.397)	2.19(.536)	6.403	−.298	74	.766

*M* Mean score; *SD* Standard deviation

Table 6 describes the results for independent t-test conducted to test the significant difference between male and female and; those who attended and did not attend training in interprofessional learning. There was no significant difference in interprofessional learning aspects between male (M = 2.33, SD = .319) and female (M = 2.31, SD = .320); t (74) = .254, *p* = 0.800. However, a significant difference was found between the participants (M = 2.16, SD = .326) and non-participants (Mean = 2.38, SD = 0.298) of IPL sessions; t (74) = − 2.698, *p* = .009. However, the IPL training non-participants scored slightly higher mean scores compared to the participants. This may be influenced by collaborative working experiences among doctors and nurses and contributed to the interprofessional attitude and perception scores.

**Table 6:** Independent t-test on demographic factors in interprofessional learning among doctors and nurses

Demographic Factor	F	t	df	*p*-value
Gender	.038	.254	74	.800
Attend sessions	.174	−2.698	74	.009

*M* Mean score; *SD* Standard deviation

One-way Anova was conducted to test the significant difference by age, highest educational qualifications, and years in practice in Interprofessional learning aspects (Table 7). There were no significant differences in interprofessional learning aspects by age, F (3, 72) = 0.769, *p* > 0.05. Similar results were revealed for highest qualification, F (2, 73) = 0.120, *p* > 0.05 and years in practice, F (3, 72) = .508, *p* > 0.05.

**Table 7:** Analysis of Variance (ANOVA) in the interprofessional learning (IPL) aspects by demographic factors

Demography Factor		Sum of Squares	Degree of freedom	Mean Square	F Value	Significant
Age	Between Groups	.235	3	.078	.769	.515
	Within Groups	7.351	72	.102		
	Total	7.587	75			
Highest qualification	BetweenGroups	.025	2	.012	.120	.887
	Within Groups	7.562	73	.104		
	Total	7.587	75			
Years in practice	Between Groups	.157	3	.052	.508	.678
	Within Groups	7.429	72	.103		
	Total	7.587	75			

## Discussion

This study shows doctors and nurses differ in their communication skills although they are required to work collaboratively. Doctors showed a higher inclination towards communication skills compared to nurses through learning with each other. Nurses preferred to stay quiet when other healthcare staff in a group express the opinion of disagreement. Communication among health professionals is mandatory to deliver safe healthcare and the inequality shown could cause serious consequences, particularly when healthcare providers like nurses are reluctant to express opinions.

In this study, an approximately equal number of doctors and nurses showed agreement to the existence of the status hierarchy in health care systems that affects relationships between doctors and nursing. This finding is consistent with the previous study by Weller et al. stating that junior doctors and nurses expressed mutual respect. However, the junior doctors feel organisational structures such as hierarchy which explicitly requires them to follow seniors often limited the extent to which they could establish trust and effective relationships with nurses [11].

Respect for each discipline is much needed by all doctors and nurses. There exist verbal outbursts by surgeons such as blaming, yelling, or threatening, causing frustration and feelings of incompetence on nursing staff. The personal attacks made by surgeons also created a strong emotional response from the nurses and would cause nurses to resign their positions or to take steps to avoid working with a particular surgeon again [28]. However, in this study, more nurses agreed that they had equal respect in each discipline they worked. It is depicted in previous studies that doctors and nurses need to portray mutual respect to avoid impairment in collaboration at work.

This study found the majority of the nurses have a positive attitude on self-assessment of communication and teamwork skills as compared to doctors. Though there were no significant differences in all the IPL aspects related to age and years of working experience, some of the nurses are from the older age group and with more years of working experience compared to doctors. The nurses’ maturity level could have contributed to their higher scores over doctors. Similarly, Pollard et al. reported maturity of participants also plays an important role as the level of confidence among matured learners was higher compared to younger students about their communication and teamwork skills [22].

In the IPL dimension, most of the doctors recorded positive attitude scores compared to nurses. Consistent with previous findings, the strongly positive scores concerning the IPL dimension, may reflect the awareness of the need for strategies that can enhance interprofessional collaboration [22]. However, in the IPI dimension, both doctors and nurses were less positive. A previous study showed that the negative shift in interprofessional interaction may be attributable to the experience of poor interprofessional interaction in work settings [22].

In the IPR dimension, doctors revealed higher positive responses compared to nurses inconsistent with the previous findings in the IPR dimension. Doctors and nurses may also be more aware of the importance of teamwork for the benefit of patients and sufficiently motivated to develop positive working relationships in practice [22]. The positive self-assessment, attitude, and negative scores found are encouraging. However, neutral and negative scores were still found, and improvement is needed as identified, poor interprofessional collaboration (IPC) can negatively affect the delivery of patient care and other health services. Interventions that address interprofessional collaborative problems have the potential to improve healthcare outcomes [29].

In this study, only the IPL attitude dimension was significantly different between doctors and nurses. There was no significant difference found between doctors and nurses in all the other three dimensions in aspects of IPL. Neutral and negatives scores are also found which requires further initiatives in interprofessional education among the health professionals in the studied hospital. Gambino et al. stated initiatives on IPL sessions are expected resulting in contacts made during the learning sessions among health professionals. These sessions have a huge potential to lead to discussions needed to resolve issues pertaining to IPL and the development of formal interprofessional education [16]. Interprofessional learning experience imparts appreciation and respect towards each other’s perspectives in the learning stage and avoids the growth of stereotyped behaviour among health professionals [30].

There was a significant difference between participants and non-participants on IPL training towards attitude and perception towards IPL. Hence, a slightly higher mean score among those non-participants of IPL training could result from the collaborative working experiences. More doctors revealed they lack confidence when they work with nurses. Pollard et al. reported maturity, prior experiences of health care, and higher education qualifications may have a breadth of experiences which, could lead to constructive discussion in interprofessional learning groups [21]. Furthermore, Pollard et al. pointed out although the IPL in the curriculum has the possibility to improve the health care delivery, the emergence of variances in responses based on a professional program proposes that IPL may not certainly influence professional socialization. Health professionals’ responses as they mature could be influenced by variables i.e. demographic and own interprofessional relationships leading to the complexity of learning [22].

### Recommendation

The results of this study indicate the need for greater incorporation of IPL in the medical and nursing curriculum. Health institutes and hospitals are encouraged to include IPL training in their routine continuous medical education. The authorities also need to allocate time for the doctors and nurses to officially participate in such training to enhance awareness of the importance of collaboration among doctors and nurses.

### Limitations

Robust evaluation of IPL requires longitudinal studies with longer follow-up periods throughout the course period [31]. This study faced difficulties in recruiting participants due to their shift work commitments and busy working schedules. Recruitments were only on a voluntary basis and obtaining the numbers in each category such as juniors and seniors were challenging.

## Conclusion

This study illuminates’ contemporary insight on self-assessment, attitude, and perception towards aspects of IPL among doctors and nurses. The gap evident from this study will contribute to the literature on IPL in clinical settings in Malaysia. Continuous formal interprofessional learning training programs have the potential in fostering positive perceptions and attitudes towards IPL. Interprofessional learning opportunities in the workplace will create potential benefits for collaborative practices. The findings of this small study revealed health professionals with IPL training showed a better self-assessment, attitude, and perception towards collaborative practices.

## Data Availability

The data and materials are not available as this study findings are based on baseline data collected in a doctoral project and need to be submitted to Universiti Putra Malaysia. However, the corresponding author can be contacted on reasonable request.
